# Controllable Formation and Real-Time Characterization of Single Microdroplets Using Optical Tweezers

**DOI:** 10.3390/mi13101693

**Published:** 2022-10-08

**Authors:** Shuai Li, Hanlin Zhang, Wenqiang Li, Yizhou Zhang, Xiaowen Gao, Haiqing Liu, Nan Li, Huizhu Hu

**Affiliations:** 1 Quantum Sensing Center, Zhejiang Lab., Hangzhou 310000, China; 2 State Key Laboratory of Modern Optical Instrumentation, College of Optical Science and Engineering, Zhejiang University, Hangzhou 310027, China; 3 Isvision (Hangzhou) Technology Co., Ltd., Hangzhou 310052, China

**Keywords:** optical tweezers, microdroplets, single, controllable formation, real-time characterization

## Abstract

Existing preparation methods for microdroplets usually require offline measurements to characterize single microdroplets. Here, we report an optical method used to facilitate the controllable formation and real-time characterization of single microdroplets. The optical tweezer technique was used to capture and form a microdroplet at the center of the trap. The controllable growth and real-time characterization of the microdroplet was realized, respectively, by adjusting experimental parameters and by resolving the Raman spectra by fitting Mie scattering to the spike positions of the spectra during the controllable growth of microdroplets. The proposed method can be potentially applied in optical microlenses and virus detection.

## 1. Introduction

Microdroplets are widely used in the fields of biology, chemistry, medicine, and material science [1,2,3,4,5,6,7,8]. Naturally occurring lipid microdroplets in cells can be used as optical microlenses to facilitate the dynamic imaging of subcellular structures and image different parts of the cell by combining light manipulation techniques to control the spatial position of the microlenses within the cell [9]. The reaction rates of common organic microchemical reactions can be increased by one to six orders of magnitude in aqueous microdroplets, in comparison with bulk solutions, under the action of an electric field [10]. Janus microparticles with different characteristics can be conveniently generated and regulated in a droplet microfluidic system, which can reduce preparation time and reagent consumption [11]. Additionally, drug-loaded nanodroplets can be used to enhance and localize drug delivery with sub-millimeter precision, which offers increased stability and prolonged half-life during circulation when compared to microbubbles [12]. The aforementioned studies verify that microdroplet quality is crucial for the effectiveness of microdroplet applications. 

Although conventional microdroplet preparation methods are currently used in several fields [13,14,15,16], verifying the stability, uniformity, and monodispersity of microdroplets is difficult. The expansion of microdroplet applications has led to higher requirements, such as the controllable growth of microdroplets and the real-time characterization of microdroplets. Among the emerging microdroplet preparation methods [17,18,19,20,21,22,23], droplet-based microfluidic technology [24,25,26,27] is considered a universal tool for widespread applications. However, despite the extensive applications and formation methods of microdroplets, existing microdroplet preparation methods generally require offline measurements for their characterization. Typically, scanning electron microscopy and transmission electron microscopy [28,29,30] are used to take measurements. Despite the high measurement accuracy, these instruments are expensive, complex to operate, and (due to offline measurements) can distort the chemical composition of microdroplets and the low timeliness of measurements. Therefore, a method that can realize the controllable formation and real-time characterization of microdroplets is ideal for the expansion of versatile microdroplet applications in various fields. It is well known that optical tweezers [31,32,33,34] are a precise tool in micro-manipulating [35,36], they may become effective method.

In this study, we propose an optical tweezer (OT)-based method to realize the controllable formation and real-time characterization of single-microdroplet. The solution used comprises aqueous sodium chloride (NaCl), aqueous potassium chloride (KCl), and aqueous disodium hydrogen phosphate (Na_2_HPO_4_) solutions. A microdroplet is trapped and formed in the focus area with a size of several to tens of microns in diameter under the effect of an optical trapping force. The growth rate and size of the microdroplet are associated with factors such as laser trapping power, solution composition, and ambient relative humidity (RH), which can realize the controllable formation of microdroplets. In addition, the parameters (such as radius and refractive index) of the microdroplets can be precisely characterized in real time using the Raman spectra. The microdroplet online controllable formation and real-time characterization method can pave the way for novel ideas and research methods in terms of super-resolution microscopic imaging and virus detection in atmospheric environments.

## 2. Materials and Methods

### 2.1. Optical Tweezers

**Figure 1:**
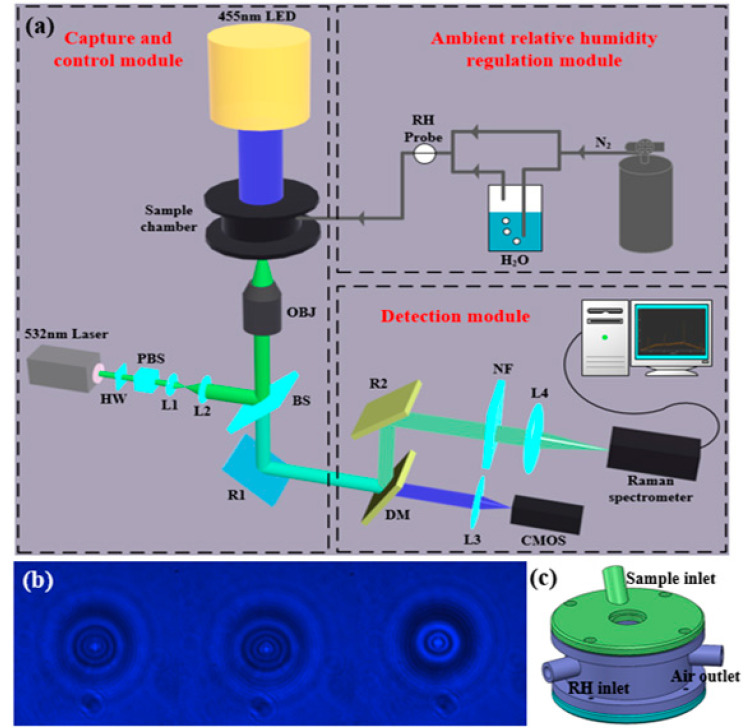
(**a**) Schematic of the microdroplet formation system based on optical tweezers (OTs). HW: half-wave plate; PBS: polarizing beam splitter; L: lens; BS: beam splitter; OBJ: objective; R: reflector; DM: dichroic mirror; NF: notch filter; LED: light-emitting diode; CMOS: complementary metal-oxide semiconductor; RH: relative humidity; N2: nitrogen. (**b**) Photographs of microdroplets captured by optical trap are taken by the CMOS. (**c**) Schematic diagram of the sample chamber.

Figure 1a illustrates the schematic of the OT-based microdroplet formation system. The system comprises a microdroplet capture and control module, an ambient RH regulation module, and a detection module. The stable capture and generation of microdroplets were both achieved with the microdroplet capture module. A sample chamber was used to provide a chamber environment when capturing the microdroplet. The ambient RH regulation module was used to adjust the RH of the sample chamber environment. The capture and generation processes of the microdroplet using OT was imaged using a complementary metal-oxide semiconductor (CMOS) in the detection module. The characteristic information of the microdroplets was detected and resolved in real time using the detection module.

As indicated in Figure 1a, a 532 nm linearly polarized light beam (Sprout-D-5W-NET, Lighthouse Photonics, 5 W, solid-state laser) was used as the laser source. The laser power was adjusted by placing a half-wave plate (HW) in front of the polarization beam splitter (PBS). A computer-controlled motorized holder was used to rotate the HW and change the output power. The adjusted light beam was brought into a high-numerical-aperture microscope objective lens (NA = 0.9, 100×, Nikon) using a telescope and beam splitter (BS); this generated the position-fixed trapping. The sample chamber was placed on a piezo-actuated translational stage. Subsequently, the transmitted light was separated by the BS and dichroic mirror (DM). To further perform the Raman spectroscopy measurements, an optical filter and a Raman spectrometer (Shamrock 750, Andor Technology) were used to monitor the beam. The backscattered Raman signal from the microdroplet was focused onto the entrance slit of the spectrometer equipped with a 1200 grooves/mm diffraction grating and an electron-multiplying charged coupled device array of 1600 × 200 pixels. A 455 nm light-emitting diode (LED) was used as the light source, and Kohler illumination was realized for bright-field imaging of the microdroplet. Images of the microdroplets were acquired using a CMOS (STC-MCA5MUSB3, 14fps, Sentech). Figure 1b shows the photographs of microdroplets captured by optical trap, which were taken by the CMOS. Figure 1c shows the schematic diagram of the sample chamber, which consists of a sample atomization inlet and a RH adjustment inlet, as well as an air outlet.

### 2.2. Method

The method for the controllable formation and real-time characterization of single microdroplets can be summarized as follows. Firstly, the laser was turned on and a laser trapping power of 60 mW was used to increase the trapping force. Secondly, a nebulizer (Omron, NE-U22V) was used to nebulize the aqueous NaCl, KCl, and Na_2_HPO_4_ solution into the sample chamber, making the NaCl, KCl, and Na_2_HPO_4_ microdroplets float in the sample chamber. Thirdly, when a single microdroplet was captured and generated at the center of the trap under the trapping force, the laser trapping power was reduced to 20–35 mW. Fourthly, experimental parameters, such as the ambient RH, laser trapping power, and solution composition, were regulated to realize the controllable formation of single microdroplets. The ambient RH in the sample chamber was regulated by introducing nitrogen gas stream into the chamber, which was realized by adjusting the ratio of dry nitrogen to wet nitrogen gas flow. Finally, during the controllable formation of single microdroplets, the back-scattered Raman signal from the microdroplet was focused onto the entrance slit of the spectrometer equipped with a 1200 grooves per mm diffraction grating and the integration time was 1s. Raman spectra data were automatically saved in a folder for real-time characterization. When Raman spectrum of the microdroplet was saved in the folder, the computer program began to solve the Raman spectroscopy data and displayed the results (radius and refractive index) in real time on the graphical user interface of the computer. The unique type of resonance exhibited during the inelastic scattering induced by the microdroplets is referred to as the whispering gallery mode (WGM). When the wavelength of light matches the resonance mode of the cavity, two counter-propagating waves form a standing wave around the microdroplet. The Mie scattering theory provides analytical descriptions of the intensity of the electromagnetic field scattered by the microdroplet [37]. The Mie scattering coefficients, namely $a_{n}$ and $b_{n}$, often referred to as partial wave amplitudes, were used to calculate the intensity of the scattered field. $a_{n}$ and $b_{n}$ are associated with the transfer magnetic and electric modes, respectively, and can be evaluated by applying the boundary conditions of the dielectric sphere. Here, we consider the microdroplet as a dielectric sphere. Then, the results were obtained as follows:

(1) $$\begin{array}{l} a_{n}(x,m)=\frac{m\psi_{n}(mx)\psi_{n}^{\prime }(x)-\psi_{n}(x)\psi_{n}^{\prime }(mx)}{m\psi_{n}(mx)\xi_{n}^{\prime }(x)-\xi_{n}(x)\psi_{n}^{\prime }(mx)}\\ b_{n}(x,m)=\frac{\psi_{n}(mx)\psi_{n}^{\prime }(x)-m\psi_{n}(x)\psi_{n}^{\prime }(mx)}{\psi_{n}(mx)\xi_{n}^{\prime }(x)-m\xi_{n}(x)\psi_{n}^{\prime }(mx)}, \end{array}$$

where m denotes the refractive index of the microdroplet relative to the surrounding medium; $x=\frac{2\pi a}{\lambda }$indicates the size parameter function corresponding to the vacuum wavelength λ; $\psi_{n}$ and $\xi_{n}$ represent the Riccati–Bessel functions of the first and second order n, respectively, which can be defined as

(2) $$\begin{array}{l} \psi_{n}(x)=xj_{n}(x)\\ \xi_{n}(x)=xh_{n}^{\left(1\right)}(x), \end{array}$$

where $j_{n}(x)$ and $h_{n}^{\left(1\right)}(x)$ denote the spherical Bessel and spherical Hankel functions, respectively, of the first order n. Alternatively, the coefficients can be expressed as

(3) $$\begin{array}{l} a_{n}(x,m)=\frac{A_{n}(x,m)}{A_{n}(x,m)+iC_{n}(x,m)}\\ b_{n}(x,m)=\frac{B_{n}(x,m)}{B_{n}(x,m)+iD_{n}(x,m)}, \end{array}$$

with

(4) $$\begin{array}{l} A_{n}(x,m)=\psi_{n}(x)\psi_{n}^{\prime }(mx)-m\psi_{n}(mx)\psi_{n}^{\prime }(x)\\ B_{n}(x,m)=m\psi_{n}(x)\psi_{n}^{\prime }(mx)-\psi_{n}(mx)\psi_{n}^{\prime }(x)\\ C_{n}(x,m)=\xi_{n}(x)\psi_{n}^{\prime }(mx)-m\psi_{n}(mx)\xi_{n}^{\prime }(x)\\ D_{n}(x,m)=m\xi_{n}(x)\psi_{n}^{\prime }(mx)-\psi_{n}(mx)\xi_{n}^{\prime }(x). \end{array}$$

For a spherical microdroplet with a real refractive index, Mie resonances occur when the imaginary parts of the coefficients $a_{n}$ and $b_{n}$ are eliminated and the real parts are equal to unity. The exact locations of these resonances can be calculated using $C_{n}(x,m)=0$ or $D_{n}(x,m)=0$. The aforementioned equations can be solved by obtaining an infinite series of roots for each mode number of a resonance. The first and second roots are labeled l=1 and l=2, respectively, where l denotes the mode order of the resonance. For a fixed mode number n, the spectral width of the resonance peaks increases with the increase in l. Conversely, for a fixed mode order l, the spectral width of the resonance peaks decreases as n increases. 

Accurate sizing of the microdroplet requires the experimental Raman spectroscopy data to be fitted to the theoretical positions of Mie scattering. Typically, the wavelengths of WGMs are calculated using the Mie scattering theory over ranges of plausible microdroplet size and refractive index, which are then compared to the experimental wavelengths. A figure of merit is assigned to each trial combination of size and refractive index that facilitates the identification of the best-fit values. 

## 3. Results and Discussion

**Figure 2:**
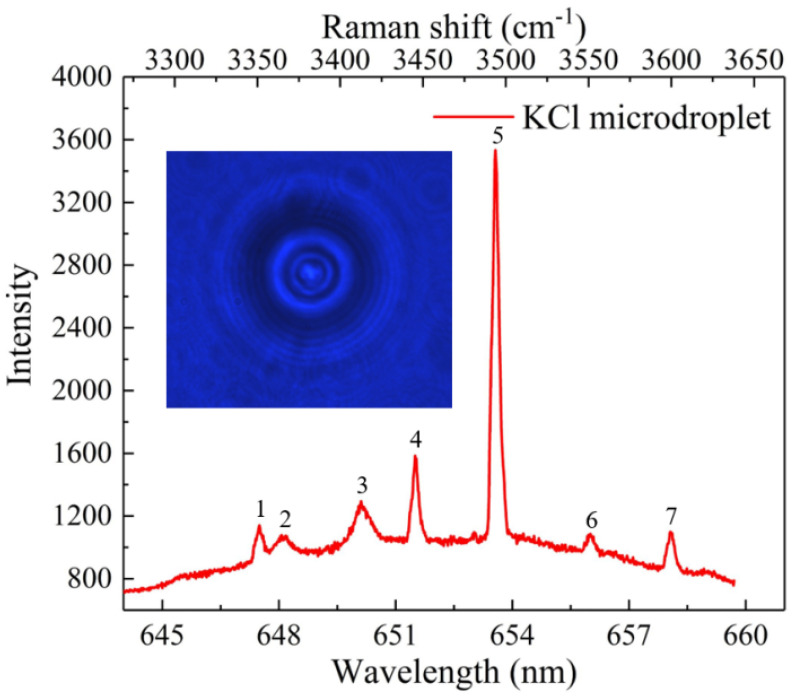
Representative morphology of a KCl microdroplet trapped by the optical tweezers (OTs) and the corresponding Raman spectroscopy signal.

We first investigated the feasibility of using OT to facilitate the formation of single microdroplets. When the laser was turned on and the OT trap existed in the sample chamber, the aqueous KCl solution (50 g/L) was nebulized into the sample chamber (RH was 60%) using an ultrasonic nebulizer at a 30° nebulization angle. A single KCl microdroplet was trapped and formed at the center of the trap in the presence of an optical trapping force. After the single KCl microdroplet was stably trapped, the Raman signal of the microdroplet was excited and collected using the Raman spectrometer. Figure 2 depicts the morphology of the KCl microdroplet trapped by the OT and the corresponding Raman spectroscopy signal with an integration time of 1 s. The Raman spectroscopy signal of the KCl microdroplet was divided into two parts. One part indicates the spontaneous Raman signal of the broadband in the range of 3300–3650 cm^−1^, which corresponds to the O-H stretching vibration of water. The other part indicates the seven sharp peaks (numbers 1–7) above the broad water band. Because the microdroplet was in a baseless suspended state that is equivalent to a spherical cavity, the laser underwent multiple total reflections inside the microdroplet. During this process, certain specific locations of the signal were significantly enhanced, generating seven sharp peaks above the broad water band. These spikes are referred to as the excited Raman peaks. The locations and intensities of the spikes were closely associated with the size of the microdroplet and its internal components. The time-resolved size and refractive index of the microdroplet can be obtained by fitting the Mie scattering to the spike positions and the fit results in the KCl microdroplet with a radius of 4.92 µm and a refractive index of 1.323. 

The principle of the formation of KCl microdroplets based on optical tweezers is as follows: after the laser was turned on, an optical trap was formed. A ultrasonic nebulizer was used to nebulize the aqueous KCl solution into the sample chamber, making the KCl microdroplets float in the sample chamber. As the refractive index of the KCl microdroplet was higher than that of the air in the surrounding environment, the higher refractive index KCl microdroplet could be captured and formed in the focus area under the trapping force. The microdroplet could grow larger at the initial stage before the changes of the RH of the sample chamber because several KCl microdroplets could be continuously captured by the optical trap. 

**Figure 3:**
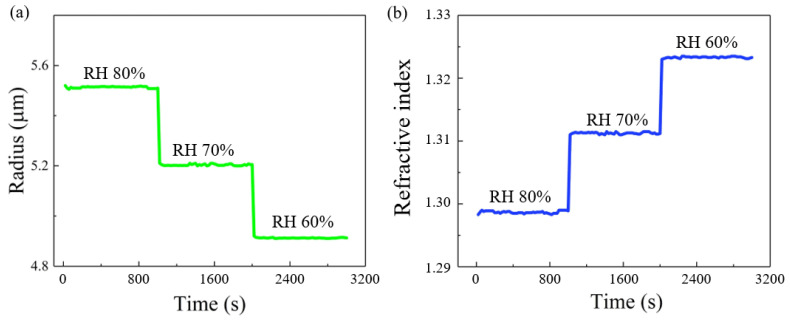
(**a**) KCl microdroplet radius and (**b**) refractive index as functions of irradiation time under different ambient relative humidity (RH) conditions.

To realize the controllable formation and real-time characterization of single microdroplets, the effects of the experimental parameters on the microdroplet growth were investigated. Initially, we investigated the effect of ambient RH on microdroplet growth. Considering an aqueous KCl solution with a concentration of 50 g/L, Figure 3a depicts the KCl microdroplet radius as a function of irradiation time under the condition that the ambient RH decreases from 80% to 60% at 10% intervals. The ambient RH was measured at a position shown in Figure 1. Depending on the flow control ratio between dry and wet nitrogen flow, it normally takes about 1-2 minutes to reduce the ambient RH from 80% to roughly 70%. It then takes about 4–5 minutes to make sure the ambient RH is maintained at a relatively stable value by fine-tuning one of the nitrogen flows. The data presented in the paper were obtained after the ambient RH was stabilized. Figure 3b shows the KCl microdroplet refractive index as a function of irradiation time under the condition that the RH decreases from 80% to 60% at 10% intervals. The obtained experimental results indicate that the variation trends of the KCl microdroplet size and refractive index are identical and opposite to that of the ambient RH, respectively. This is because when the ambient RH decreases, the KCl microdroplet evaporates water and reduces the microdroplet size, which in turn increases the refractive index.

**Figure 4:**
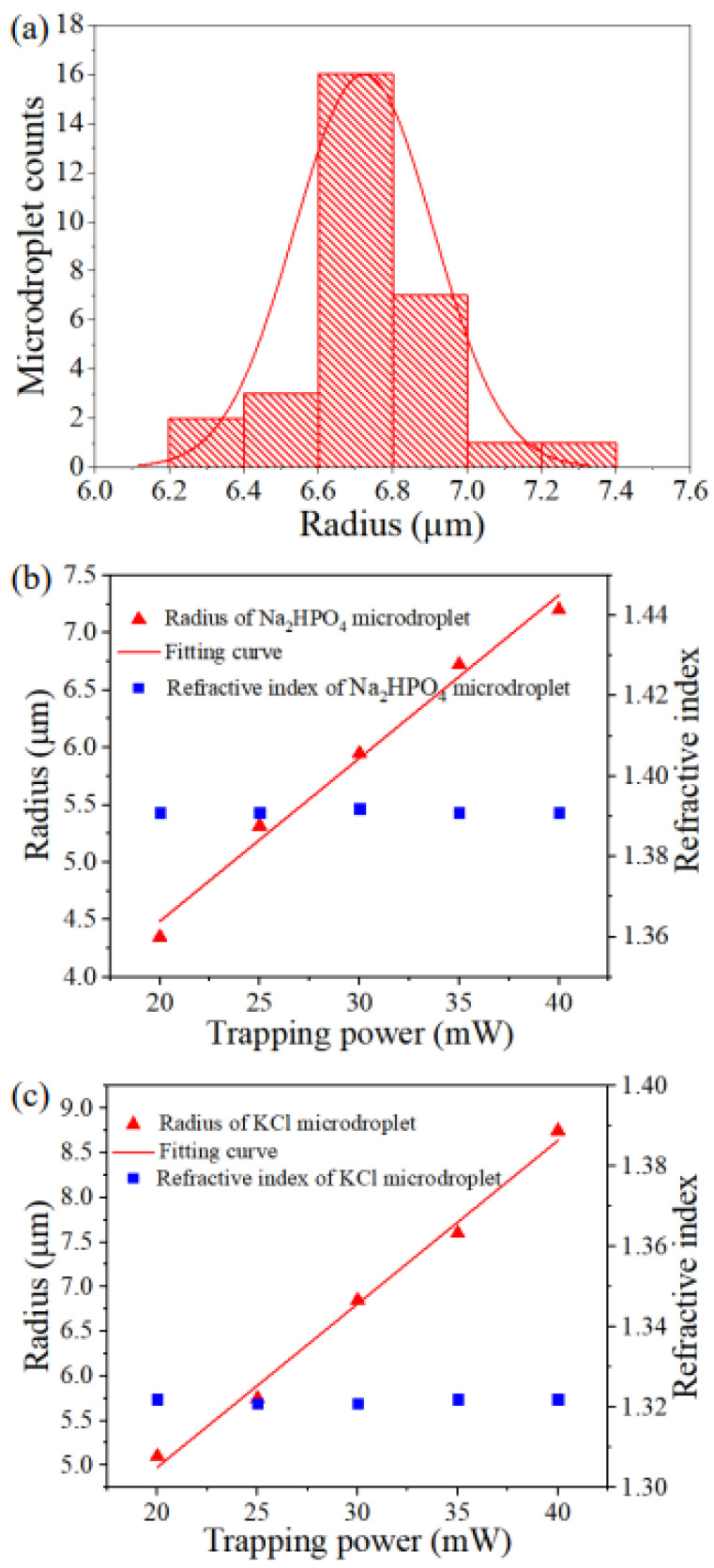
(**a**) Histogram of the radius of the Na_2_HPO_4_ microdroplet. (**b**) The Na_2_HPO_4_ microdroplet and (**c**) The KCl microdroplet radius and refractive index as a function of the trapping power ob-tained using the same method of the radius histogram in (**a**). The solid line denotes the linear fitting curve.

Subsequently, the effect of laser trapping power on microdroplet growth was investigated. In the case of Na_2_HPO_4_ solution with a concentration of 122 g/L and KCl solution with a concentration of 50 g/L, the ambient RH was 70%, and the laser trapping power was 35 mW. Figure 4a shows the radius histogram of the Na_2_HPO_4_ microdroplet under a laser irradiation time of 600 s. To investigate the effect of laser trapping power on Na_2_HPO_4_ microdroplet growth, the radius histogram was transformed into a mean radius value versus the trapping power. Figure 4b depicts the Na_2_HPO_4_ microdroplet radius as a function of the laser trapping power under an ambient RH of 70% and a laser irradiation time of 600 s. Figure 4c depicts the KCl microdroplet radius as a function of the laser trapping power under an ambient RH of 70% and a laser irradiation time of 600 s. The growth rate of Na_2_HPO_4_ and KCl microdroplets were almost positively proportional to the laser trapping power. This phenomenon can be attributed to the fact that the higher the laser trapping power, the higher the optical trapping force, which can trap multiple microdroplets at the center of the OT trap. As the microdroplets are liquid, multiple microdroplets can fuse into one large microdroplet. The refractive index of Na_2_HPO_4_ and KCl microdroplets remains almost constant because the ambient RH in the sample chamber is constant and no moisture absorption or volatilization of the microdroplets occurs.

**Figure 5:**
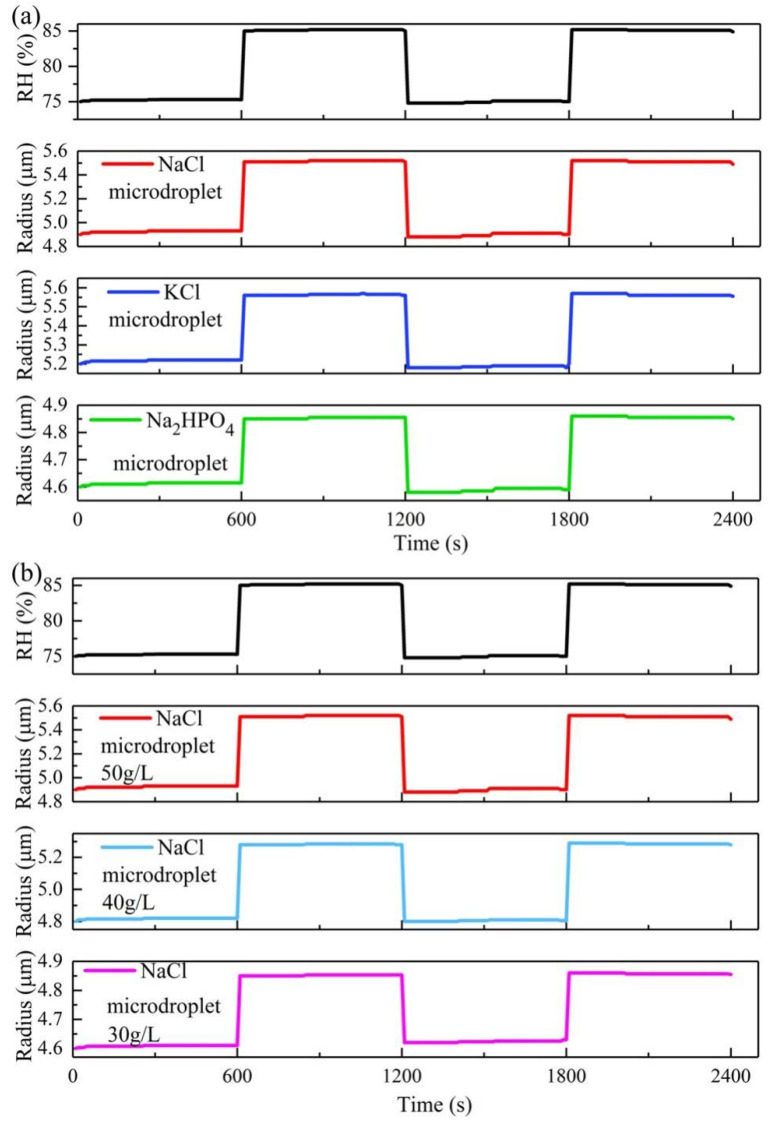
(**a**) Growth of different types of microdroplets and (**b**) NaCl microdroplets of different concentrations under the conditions that the ambient relative humidity (RH) changes between 75 and 85%.

Finally, the effect of the solution composition on the microdroplet growth was analyzed. The laser trapping power was 20 mW. We considered the aqueous NaCl, KCl, and Na_2_HPO_4_ solutions with concentrations of 50 g/L. Figure 5a shows these three types of microdroplet radius as a function of irradiation time under ambient RH changing between 75% and 85%. As indicated in Figure 5a, the NaCl microdroplet exhibits the fastest growth rate, followed by the KCl microdroplet; the Na_2_HPO_4_ microdroplet has the slowest growth rate owing to the activity of hydrophilic ions in different microdroplets. Figure 5b depicts the growth of different concentrations of NaCl microdroplets when the ambient RH varies between 75% and 85%. Evidently, the higher the concentration, the greater the ability of the NaCl microdroplet to absorb water and the larger the corresponding NaCl microdroplet size. This is consistent with the phenomenon where the highest hygroscopicity was identified at the highest concentration of NaCl in the moisture absorption tests [38].

The results obtained from the above experiments verify that a controllable growth of single microdroplets can be achieved by adjusting the ambient RH, laser trapping power, and solution composition. Additionally, a real-time characterization of single microdroplets can be realized by fitting the Mie scattering to the spike positions of the Raman spectra.

## 4. Conclusions

In conclusion, we demonstrated that the controllable formation and real-time characterization of single microdroplets could be realized using the OT technique. Initially, microdroplet was formed at the center of the OT trap. Subsequently, the controllable growth of single microdroplets was realized by adjusting the ambient RH, laser power, and solution composition. At the end, the real-time characterization of parameters, including the radius and refractive index of single microdroplets, was realized by fitting the Mie scattering to the spike positions of the Raman spectra. With advantages such as the controllable formation and real-time characterization of single microdroplets, such OT-based microdroplet formation and characterization methods can find potential applications in optical microlenses and virus detection, such as optical microdroplets for super-resolution microscopic imaging and antibody drug microdroplets for virus detection in atmospheric environment.

In our future work, we will investigate optical super-resolution imaging based on microdroplets generated and characterized by optical tweezers. By adjusting the composition and diameters of the microdroplets, it is possible to improve the super-resolution imaging ability. Since microdroplets are non-toxic and harmless and do not damage the microstructure to be measured, it is possible to achieve a low-cost, non-destructive, and real-time observation of microstructures smaller than 200 nm and break the diffraction limit. However, to realize the optical super-resolution imaging using microsroplets generated by optical tweezers, the microdroplets can be flexibly moved to the microstructures. To solve this problem, an electro-optical component or an acousto-optical component should be added to the optical path to move the microdroplets generated by the optical trap. We believe that with unremitting efforts, we will have good results in achieving super-resolution imaging based on microdroplets generated and characterized by optical tweezers.

## Data Availability

Not applicable.
